# Characterization of bacterial community and flavor differences of different types of Douchi

**DOI:** 10.1002/fsn3.2280

**Published:** 2021-05-18

**Authors:** Yurong Wang, Fanshu Xiang, Zhendong Zhang, Qiangchuan Hou, Zhuang Guo

**Affiliations:** ^1^ Hubei Provincial Engineering and Technology Research Center for Food Ingredients Hubei University of Arts and Science Xiangyang China

**Keywords:** bacterial diversity, Douchi, flavor, functional prediction

## Abstract

According to the appearance and technology, traditional fermented Douchi can be divided into dried Douchi and wet Douchi. However, there are few reports on the difference of bacterial community structure between them or the influence of bacterial community on product flavor. In this study, high‐throughput sequencing technology and electronic nose were used to measure the bacterial diversity and flavor of 40 Douchi samples, and the correlation between them was explored by multivariate statistical means combined with COG database. Results showed that the cumulative average relative abundance of Firmicutes and Proteobacteria in the samples was as high as 95.93%, and the former was the core bacteria phylum. On the whole, the dominant bacteria in Douchi were *Bacillus* (50.67%), *Staphylococcus* (14.07%), *Enterococcus* (2.54%), *Proteus* (1.61%), *Brevibacillus* (1.46%), *Providencia* (1.26%), *Weissella* (1.24%), and *Ureibacillus* (1.19%). LEfSe analysis indicated that *Bacillus* can be used as a biomarker in dried fermented soybeans. Meanwhile, dried samples contained more intensive aromatic substances, but were significantly lower in W6S (selectivity to hydrogen) and W3S (methane‐aliph) compared with the wet samples. *Aneurinibacillus* and *Brevibacillus* were helpful to the formation of aromatic flavor in Douchi, but *Vagococcus* and *Corynebacterium* were the opposite. Gene and microbial phenotypic prediction showed that microorganisms in dried Douchi use protein more efficiently, while in wet Douchi, microbial energy metabolism was more vigorous. The pathogenic potential of microorganisms in dried samples was higher than that in wet. This study can sound the alarm for improving the safety of home‐brewed Douchi and provide guidance for the subsequent screening of strains that enhance the flavor of fermented soybeans.

## Graphical abstract

Bacillus, Staphylococcus, Enterococcus, Proteus, Brevibacillus, Providencia, Weissella, and Ureibacillus were the dominant bacteria of Douchi; Brevibacillus and Aneurinibacillus had significant positive correlations with three aromatic characteristics but Vagococcus and Corynebacterium were the opposite; Microorganisms in dried Douchi use protein more efficiently, while in wet Douchi, microbial energy metabolism was more vigorous.

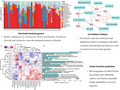

## 1 INTRODUCTION

As a traditional fermented bean product, Douchi is widely used, which can be used not only as main course but also as seasoning. In addition to supplying flavor and protein (Chen, 2012), it can inhibit breast (Messina et al., 1994) and prostate (Wang et al., 2008) to some extent. Studies have shown that Douchi can also treat diseases such as indigestion (Chen, Wang, et al., 2011; Chen, Xiong, et al., 2011) and restlessness (Jing et al., 2007). In China, the traditional production process of fermented soybeans mainly includes selecting soybeans, cleaning, steaming, cooling, adding salt, peppers, and other auxiliary materials. Dried fermented soybeans need to be spread outdoors and dried until they do not stick, while wet fermented soybeans will be added with boiled and cooled soybean soup. The last step is carried in a closed tank involving many natural microorganisms (Tamang et al., 2016). And the small amount of air contained in the tank at the early stage permits the growth and reproduction of aerobic bacteria. Moreover, since the fermentation is performed in a relatively uncontrolled manner with various microbes existing in the air, tank, water, and raw materials, a complex bacterial community structure is formed in the final product. This complex natural microbial flora has a strong influence on the physical properties, sensory quality, and flavor characteristics of Douchi (Chen et al., 2017). At present, the researches on Douchi mainly focus on the screening of excellent strains (Guo et al., 2019) and the optimization of production process (Deng et al., 2015). However, the microbial diversity, fermentation mechanism, and the influence on flavor quality are poorly understood. Therefore, this study is particularly important.

High‐throughput sequencing has been widely used in analyzing microbial community structure of various environments (Antonia, 2017), and the second‐generation technology represented by Illumina MiSeq has the advantages of fast detection and high throughput (Caporaso et al., 2012). Besides, this method can accurately identify hard‐to‐culture and low‐abundance microorganisms, providing a new tool for exploring the diversity and function of microorganisms in fermented food. Eugenia (Jiménez et al., 2017) explored the diversity of lactic acid bacteria in Peruvian traditional fermented potatoes by high‐throughput sequencing; Justyna (PolKa et al., 2015) used this technique to analyze the bacterial diversity of Italian sausages at different maturity stages; Zhang (Zhang et al., 2018) has investigated the microbial diversity of Douchi made from different raw materials. The implementation of these research topics provides a good idea for analyzing the bacterial diversity of Douchi in this study. E‐nose technology can realize rapid digital evaluation of volatile flavor substances in food. The measurement is precise and less‐affected by subjective factors (Loutfi et al., 2015), thus extensively employed in the fermented food industry (Fan et al., 2017; Jung et al., 2017).

In this study, 21 dried and 19 wet Douchi samples were collected from farmers in Enshi, Hubei Province, China. The microbial diversity was determined with Illumina MiSeq high‐throughput sequencing. Meanwhile, e‐nose system was used to detect the intensity of flavor indexes, and a correlation analysis was performed with the dominant genus. Furthermore, the function and phenotype of bacterial microbes were predicted based on PICRUSTs and BugBase. This study aims to provide a theoretical basis for the quality improvement in production and the formulation of relevant standard.

## 2 MATERIALS AND METHODS

### 2.1 Bacterial diversity analysis

#### 2.1.1 Sample collection and metagenomes extraction

This study adopted a completely randomized design. A total of 40 Douchi samples were collected local farmers' homes in Enshi Tujia and Miao Autonomous Prefecture, Hubei Province, China, including 21 dried samples (D1‐D21) and 19 wet samples (W1‐W19). The main raw material is soybean, which was planted by farmers themselves. All samples were prepared by traditional methods; that is, they were naturally fermented without adding any starter. The main difference between the two types of samples is whether added boiled soybean soup or not. Wet Douchi was fermented with enough soybean soup to immerse the soybeans, while no water was added during the fermentation of dried Douchi.

The total DNA was extracted from 3.0 g of each sample according to the instruction of food DNA Genomic Extraction Kit (QIAGEN). The purity, concentration, and integrity of the extracted DNA were detected by spectrophotometry and 1% agarose gel electrophoresis. Qualified DNA samples were stored in a −20°C refrigerator for use.

#### 2.1.2 PCR amplification and sequencing

In this study, the V_3_‐V_4_ region of 16S rRNA was amplified using forward primer 338F (5′‐ACTCCTACGGGAGGCAGCAG‐3′) and reverse 806R (5′‐GGACTACHVGGGTWTCTAAT‐3′). The forward primer was barcoded with different nucleotide sequences. The PCR amplification system included the following: 4 μl 5×PCR buffer, 2 μl 2.5 mM dNTPs mix, 0.8 μl 5 μmol/L forward primer, 0.8 μl 5 μmol/L reverse primer, 0.4 μl 5 U/μl DNA polymerase, 10 ng DNA template, supplemented to 20 μl with ddH_2_O. The PCR amplification conditions were as follows: 95°C for 3 min; 95°C for 30 s, 55°C for 30 s, 72°C for 45 s, 30 cycles; 72°C for 10 min.

The 40 qualified DNA amplicons, diluted to a concentration of 100 nmol/L, were sequenced by MiSeq high‐throughput sequencing platform in Shanghai Majorbio Co., Ltd.

#### 2.1.3 Quality control and bioinformatics analysis

The pair‐ended sequences generated through MiSeq sequencing were merged referring to the overlap relationship of paired fragment sequences. Sequences were divided into different samples by referring to the barcodes. The barcodes and primers in the aligned sequences were removed to obtain high‐quality information for further analysis. Sequences with less than 50 bp of bases after cutting were discarded.

Bacterial community analysis and diversity assessment were performed on the retained sequences using the QIIME (v1.9.1) platform (Ju & Zhang, 2015). The procedure was as follows: (a) calibrating and aligning the high‐quality sequences with PyNAST (Awasthi et al., 2017); (b) using UCLASS to cluster the aligned sequences based on 100% similarity to establish a complete representative gene set of 16S rRNA; (c) clustering the aligned sequences based on 97% similarity by UCLASS to generate operational taxonomic units (OTUs) (Hou et al., 2016); (d) identifying and deleting chimeras in the constructed OTU matrix through ChimeraSlayer (Wei et al., 2016); (e) performing homology comparison among the representative reads with RDP, Greengenes, and SILVA databases, annotating taxonomic positions at the phylum, class, order, family and genus levels, and combining annotation results generated by different databases (Balvoiūt & Huson, 2017); (f) drawing phylogenetic tree based on OTU representative sequence with FastTree (Yu et al., 2017); (g) assessing bacterial abundance and diversity in each sample basing on α diversity indexes including the observed species, Shannon index, Chao1 index and Simpson index, evaluating whether the sequencing depth meets the requirements of subsequent bioinformatics analysis with Shannon Index Curve and exponential curve of observed species; (h) leveling all samples 1,000 times randomly based on the OTU matrix data according to the number of samples containing the minimum sequences to reduce error, combining the results obtained from the 1,000 replicates and doing β diversity analysis; (i) investigating inter‐sample β diversity by Principal Co‐ordinates Analysis (PCoA) based on UniFrac and cluster analysis by the Unweighted Pair‐Group Method with Arithmetic means (UPGMA).

### 2.2 Flavor quality evaluation through e‐nose technology

Dispense 30 g Douchi sample into three e‐nose vials, heat in water bath (Yiheng) at 60°C for 15 min, let it stand at room temperature for 20 min, insert e‐nose probes into the sample, and collect volatile compounds using 10 sensitive metal sensors. Before testing, the sensors performed an automatic cleaning of 95 s, followed by 60 s sample determination. Response value was measured every second. The response curve tended to level off after 40 s of measurement. Therefore, response value at 49 s, 50 s, and 51 s was averaged to construct the data matrix. Response value was defined as the ratio of G to G0, which represented the resistance of metal sensors during sample test and the air test, respectively. The reading was 1 during automatic cleaning. The greater the value deviates from the value of 1, the higher the concentration of volatile compounds was (Zhang et al., 2014).

### 2.3 Functional and phenotypes prediction of bacterial

Quality control qualified sequences were clustered into OTU matrix by UCLASS with the identity threshold of 97% according to the standard database of PICRUSTs (Gavin et al., 2018). Taxonomic positions of representative sequences were annotated with the Greengenes database. The PICDRUSTs software was used to predict the functional potential of bacterial flora in Douchi samples. At the same time, the annotated OTU matrix was uploaded to the online website (https://bugbase.cs.umn.edu/) for microbiome phenotype prediction (Ward et al., 2017).

### 2.4 Statistical analysis

Linear discriminant analysis effect size (LEfSe) method based on a normalized relative abundance matrix was used to identify the significant differences between cheese samples (Jin et al., 2018). Pearson correlation analysis and Wilcoxon test were applied to calculate the correlation and significance between bacteria genera (with an average relative abundance of more than 0.5%) and the relative intensity of flavor indexes generated by e‐nose. Cytoscape software (v3.5.1) was used to map the correlation diagram; correlation and significance were also calculated with Pearson correlation analysis and Wilcox test between the gene function and the bacteria genera that amounted for more than 0.5% of the sequences. The result was visualized through a heat map, and all the figures were plotted by R software (v3.3.2) as well as Origin software (v2018, OriginLab Corp).

## 3 RESULTS AND DISCUSSION

### 3.1 Microbial community diversity and abundance

A total of 1,865,973 high‐quality 16S rRNA sequences were obtained from 40 Douchi samples with an average of 46,649 sequences per sample (range: 36,166–62,430, *SD* = 6,985). Based on the 97% similarity threshold, they were classified into 168,565 OTUs, 4,214 per sample (range: 1,798–7,588, *SD* = 1,381). Statistics analysis indicated the average number of reads and OTUs were 47,633 and 4,389 respectively for the 21 dried samples, 45,562 and 4,020 respectively for the 19 wet samples. More detailed 16S rRNA sequencing results were shown in the Table S1.

In this study, the observed species curve and the Shannon index curve were used to further evaluate the sequencing depth to determine whether it met the requirements of subsequent bioinformatics analysis (Figure S1a). It could be seen from the figures that both the rarefaction curve and the Shannon curve tended to be flattened with the increase of sequencing depth, indicating that the amount of data satisfied the requirements. Though the sparse curve did not plateau, the Shannon curve reached saturation, meaning that some additional new species may be discovered by expanding the coverage, but the vast majority of microbial and bacterial diversity was captured.

### 3.2 Compare bacterial distribution of different types of Douchi

**FIGURE 1:**
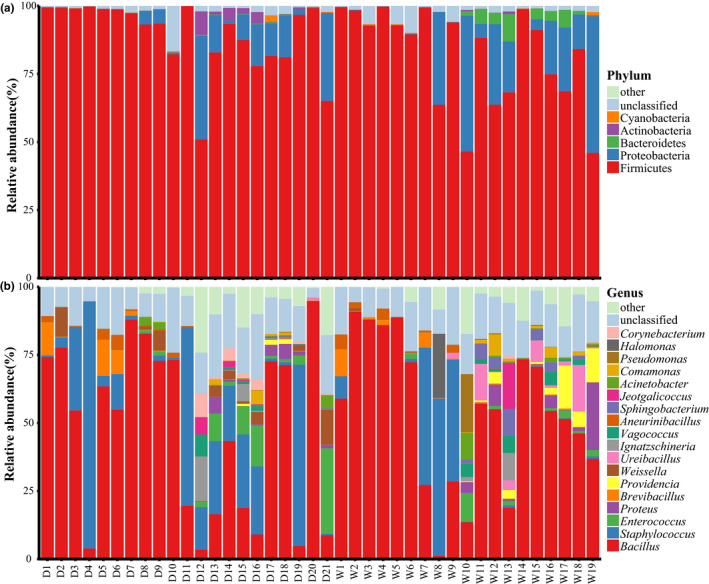
The relative abundance of Dominant phyla (a) and genera (b) from different type samples. D, dried Douchi; W, wet Douchi. The average relative abundance >0.1% was defined as dominant bacteria phyla or dominant bacteria genera

A total of 9 bacterial phyla were identified among all Douchi samples (Figure 1a), of which 5 were the major phyla (average relative abundance more than 0.1%), included Firmicutes (85.93%), Proteobacteria (10.00%), Bacteroidetes (0.96%), Actinobacteria (0.61%), and Cyanobacteria (0.16%). Firmicutes, present in the samples with relative abundance all above 46%, were the absolute dominant phyla. Secondly, Proteobacteria were also found in all samples, with lower abundance in some samples though. At the genus level, a total of 192 bacterial genera were identified (Figure 1b), of which 18 were dominant genus (average relative abundance more than 0.50%). Eight out of the eighteen were reported a relative abundance higher than 1.0%, namely *Bacillus* (50.67%), *Staphylococcus* (14.07%), *Enterococcus* (2.54%), *Proteus* (1.61%), *Brevibacillus* (1.46%), *Providencia* (1.26%), *Weissella* (1.24%), and *Ureibacillus* (1.19%). In addition, *Bacillus* and *Staphylococcus* existed in all the tested samples, making them the overwhelming dominate bacteria.

Firmicutes and Bacteroidetes are widespread in fermented food (Marco et al., 2017). Bacteroidetes abundance differs significantly between the two Douchi types. It was documented that *Flavobacteria* (phyla: Bacteroidetes) lived mainly in aquatic environment, especially in liquid fermented food (Rios‐Castillo et al., 2018). This was highly consistent with our results. We found Bacteroidetes only in wet samples, not in the 21 dried samples. At the genus level, *Bacillus* and *Staphylococcus*, two genera colonized in all samples, were key to the fermentation process (Shin & Jeong, 2015). Significance testing of dominant genus indicated that there were significant differences (*p* < .05) between the two types in terms of *Staphylococcus, Brevibacillus, Ureibacillus,* and *Pseudomonas*. Studies showed that *Bacillus* produced a large number of enzymes such as proteases (Gaurav et al., 2015) and amylases (Akhani et al., 2017), which could be used commercially for industrial production. As a result, the presence of *Bacillus* can accelerate the fermentation process of Douchi and provide substances required by other bacteria (Zhu et al., 2017). *Staphylococcus* appeared in all the samples as a dominant genus with significant differences, exhibiting good adaptability to the environment of Douchi fermentation. Previous studies have shown its proteolytic function and antibiotic activity during Douchi fermentation (Furuhata et al., 2010; Jeyaram et al., 2008), revealing its importance in the process (Tae et al., 2010). Yang (Yang et al., 2016) revealed that the relative abundances of the other predominant bacteria such as *Brevibacillus, Ureibacillus* and *Pseudomonas* were positively related to the fermentation duration, reaching the peak after 1 week of fermentation. Researches also demonstrated that *Brevibacillus* fermentation could produce glutamate (Momose & Takagi, 2014), *Ureibacillus* could decompose cellulose to butanol (Mariem et al., 2018), and most *Pseudomonas* could produce one kind or more kinds of protease, lipase, and lecithin enzymes (Dogan & Boor, 2003), thereby enhancing the flavor of the Douchi.

**FIGURE 2:**
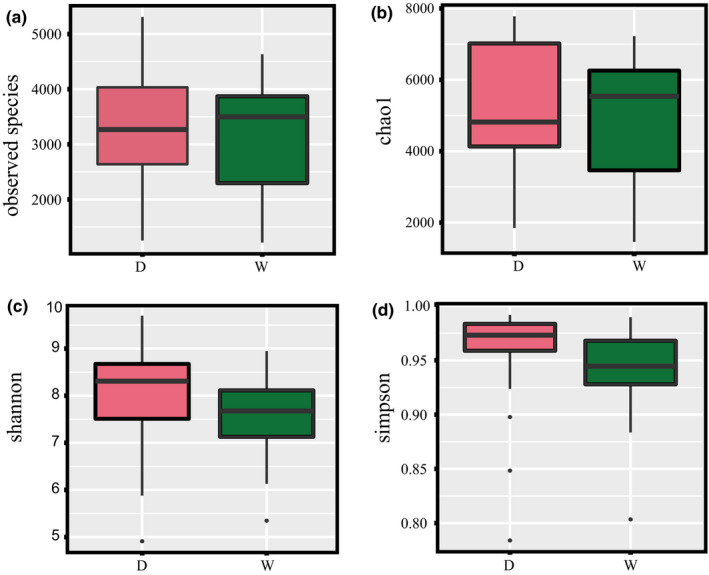
Analysis of sequencing effect based on alpha diversity indexes. (a) observed species; (b) Shannon index; (c) Chao1 index; (d) Simpson index

Microbial population datasets of all samples were compared to investigate their differences with regard to bacterial flora. The abundance and uniformity of bacterial species were characterized by the OTU grade and the number of OTU sequences as the abscissa and ordinate respectively in Rank abundance curve (Figure S1b). In terms of horizontal axis, the curve widths of the dried and wet samples were substantially the same with larger range in several samples. When it came to the vertical axis, the curves of the dried samples were smoother. This indicated some differences in the abundance between the two types, and the species distribution in the dried samples was more uniform. Then, the bacterial abundance and diversity of different types of Douchi were compared and analyzed (Figure 2). The number of observed species (Figure 2a) and the Chao1 index (Figure 2c) demonstrated that wet Douchi had higher bacterial richness. On the other hand, dried Douchi was featured with higher Shannon index and Simpson index, indicating more diversity.

**FIGURE 3:**
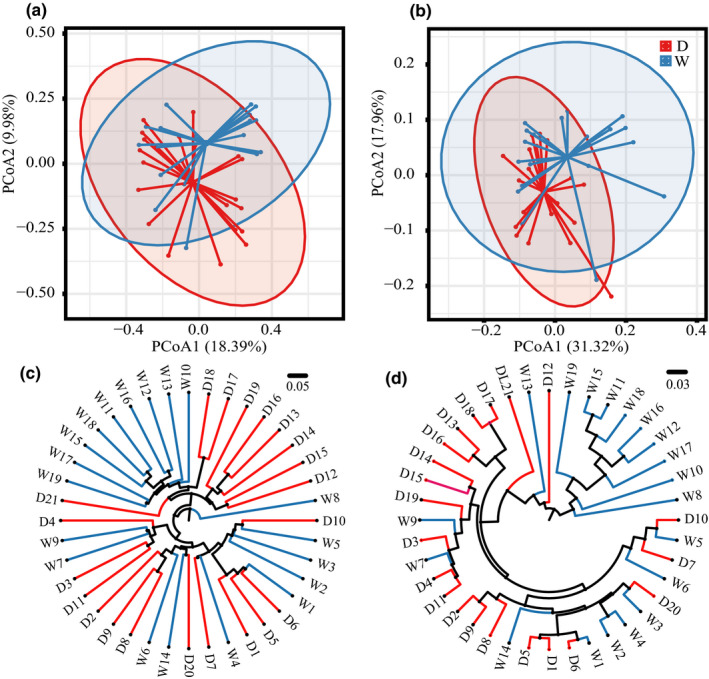
Analysis of bacterial community differences in different types of Douchi based on UniFrac distance. (a) & (b) Unweighted and Weighted PCoA; (c) & (d) unweighted and weighted cluster analysis. Red is dried fermented soybean, and blue was wet

Microbial community structure differences between the two types were mapped visually through PCoA based on UniFrac and cluster analysis by UPGMA (Figure 3). The results of PCoA displayed obvious separation tendency despite of some coincidences in the spatial arrangement of different samples. Cluster analysis by UPGMA was consistent with PCoA in results. Significant differences were proved by constrained Canonical Correspondence Analysis (CCA) and multivariate analysis of variance (MANOVA) (Figure S2), which suggested that there was significant difference (weighted analysis *p* = .003, unweighted analysis *p* = .029) in the microbial community structure between the two types of Douchi. Chen (Chen, Wang, et al., 2011; Chen, Xiong, et al., 2011) studied the microbial community in Douchi during the late phase of fermentation. Their results were the same as this study. Fermentation method posed a profound impact on the fermentation environment of Douchi, leading to divergence in the flora structure (Bortolini et al., 2016). Result showed that difference not only existed between different types, but also among the samples of the same type and the former was larger. Inter‐type differences may attribute to makers’ empirical procedure and varied standard as well as the surrounding environment even if they use the same type of fermentation (Moretro & Langsrud, 2017).

In order to further identify the differences among the abundant groups, LEfSe was performed with a threshold score of 2.0 (Figure S3). A total of 45 mycobacteria with significantly different abundance were identified in the study (*p* < .05), of which 36 and 9 were crucial to wet and dried Douchi, respectively. The LEfSe performed to further pinpoint the differential taxa in these microbiota communities, showed that the mean relative abundance of most bacterial groups in the wet samples was high. The result indicated *Rhodococcus*, *Sphingobacterium*, *Atopostipes*, *Pseudomonas*, *Rhodococcus*, *Brevibacter*, *Microbacterium*, *Psychrobacter*, *Erwinia*, *Halomonas*, *Globicatella*, *Lactobacillus*, *Stenotrophomonas*, *Acinetobacter*, *Comaminas*, *Leuconostoc*, *Bacillus*, *Wohlfahrtiimonas*, *Kocuria*, Nocardiopsaceae, *Ignatzschineria,* and *Psychrobacter* played an important role in wet Douchi and could be used as biomarkers, while *Bacillus* was found in wet fermented soybeans (Segata et al., 2016).

### 3.3 Comparative analysis of flavor quality of different types of Douchi

Following the bacterial diversity, analysis was a flavor quality assessment through e‐nose technique (Hao et al., 2017). A comparative study was done by classifying the samples into two groups according to their feature (Table 1). The results were as follows: Compared with the wet Douchi, the dried samples were higher with regard to the mean relative intensity of W1C, W5S, W3C, W5C, W1S, W1W, W2S, and W2W, though no significant difference (*p* > .05) between the two groups was detected by Wilcox test. In spite there were no significant differences, the readings of three aromatic indicators W1C, W3C, and W5C in dried samples were more intensive than those of the wet. It was interesting that the wet samples only led in W6S and W3S among the 10 flavor quality parameters and differences were significant between the two groups (*p* < .05).

**TABLE 1:** Performance description of electronic nose sensors and flavor difference analysis of two types of fermented soybeans

Electrode	Performance	D	W	*p‐value*
W1C	Aromatic	0.29 (0.33, 0.05–0.45)	0.25 (0.24, 0.07–0.42)	.279
W5S	Broadrange	20.72 (17.71, 2.20–58.62)	15.27 (9.48, 2.25–42.45)	.516
W3C	Aromatic	0.42 (0.50, 0.10–0.63)	0.39 (0.38, 0.15–0.60)	.386
W6S	Hydrogen	1.14 (1.12, 0.99–1.41)	1.99 (1.23, 1.11–10.04)	.002
W5C	Arom‐aliph	0.48 (0.56, 0.09–0.72)	0.46 (0.47, 0.17–0.69)	.481
W1S	Broad‐methane	22.28 (10.78, 5.75–97.79)	20.61 (20.20, 7.12–60.70)	.167
W1W	Sulfur‐organic	33.04 (33.82, 7.81–72.67)	28.59 (23.00, 3.36–53.92)	.569
W2S	Broad‐alcohol	9.20 (4.34, 2.45–36.60)	7.92 (6.67, 3.36–27.04)	.176
W2W	Sulph‐chlor	3.84 (3.00, 2.17–9.16)	3.20 (2.98, 1.83–5.22)	.665
W3S	Methane‐aliph	1.50 (1.37, 1.17–2.54)	2.07 (1.89, 1.30–3.88)	.002

Note

The data in the table are average (median, minimum–maximum).

**FIGURE 4:**
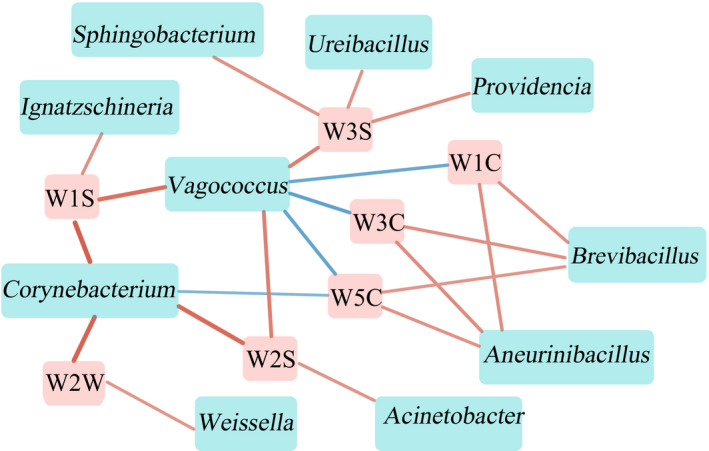
Correlation network diagram of dominant bacteria and flavor index. The blue box indicates the dominant genus and the red square indicates the flavor. The red line indicates a positive correlation, the blue line indicates a negative correlation, and the thickness of the line indicates the magnitude of the correlation

A correlation map was drawn using the dominant genus and flavor quality data (Figure 4). Ten out of 18 dominant genera were strongly correlated with 7 flavor indicators (*p* < .05). And there was positive correlation between most bacteria genus and sensor indicators, among which *Brevibacillus* and *Aneurinibacillus* had significant positive correlations with three aromatic characteristics (*p* < .05), while *Vagococcus* and *Corynebacterium* were negatively associated (*p* < .05). W1C, W3C, W5C, and W2W are excellent quality indicators mainly determining aromatic substances, and *Vagococcus* was negatively correlated with the three sensors, while *Brevibacillus* and *Aneurinibacillu* were just the opposite, and *Weissella* was positively correlated with W2W, indicating that *Vagococcus* is not conducive to the formation of Douchi flavor.

### 3.4 Functional annotation of unigenes across the COG database

**FIGURE 5:**
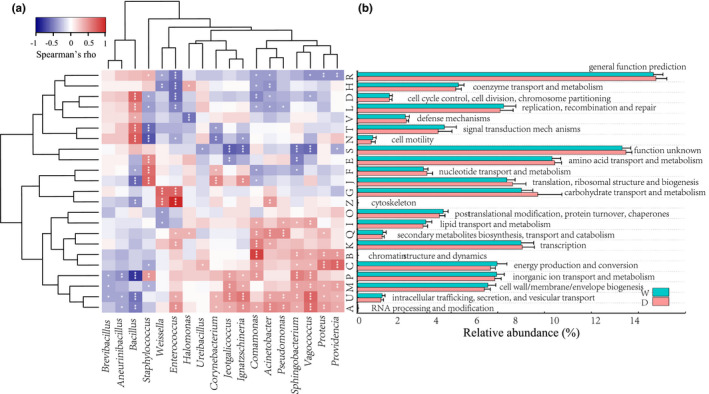
Functional annotation of Douchi bacteria based on cog database and its correlation with dominant bacteria. (a) Spearman's rank correlation between the COGs functional categories and Dominant bacteria genera. Significant correlation is represented by ***p < .001, **.001 < *p* < .01, *.01 < *p* < .05, respectively. (b) Relative abundance of protein classification of bacteria from Douchi samples

PICRUSTs gene prediction generated a total of 4,267 COGs which belonged to 23 functional categories (Figure 5b). Comparison between the two types of Douchi revealed that category E (amino acid transport and metabolism), G (carbohydrate transport and metabolism), and K (transcription) were dominant in all samples. According to Wilcox test, four out of the 23 functional categories had significant differences between the two groups (*p* < .05) were category G (carbohydrate transport and metabolism), C (energy production and conversion), O (posttranslational modification, protein turnover, and chaperones), and T (signal transduction mechanisms). There were apparently more category G sequences and less category C, O, and T in dried samples compared with the wet ones (*p* < .05).

As expected, most of the sequences were functionally assigned to genes related to energy metabolism, protein metabolism, and ion transporters. Energy and protein metabolism were essential for the survival and related functions of microorganisms. Ion transport was also indispensable to basic bacterial metabolism. Ions served as enzyme cofactors to achieve a wide variety of biochemical reactions. The dried samples overtook the wet in the amount of category G genes, indicating that the former was more efficient in protein utilization. In contrast, there were more gene sequences with potential functions of category C, O and T in wet samples. In bacteria, the generation of energy relied primarily on substrate phosphorylation, a process in which glucose was broken down into acetate (Gao et al., 2017). The fermentation of Douchi involved unique microbial metabolic pathways of carbohydrates, which produced diversified volatile short‐chain fatty acids (SCFA). Besides, high expression of genes associated with signal transduction mechanisms and others may enhance the signal sensing ability of bacteria, thereby enabling them to utilize nutrients and eliminate or avoid toxic substances better. As an essential substance in organic body, protein should be translated, modified, and converted before exerting its specific role. This suggested that the metabolism in wet Douchi may be more active. These functional categories were extremely important to the fermentation of Douchi. Their high expression showed that bacteria can improve fermentation efficiency, shorten production cycle, and increase utilization rate of raw materials.

In order to determine the potential function of single genus, this study performed a rank correlation and significance analysis for the abundance matrix of COGs functional categories and the dominant genus (Figure 5a). The analysis found that category K (transcription) and C (energy production and conversion) were basically similar in their correlation and significance to the dominant genus. Category G (carbohydrate transport and metabolism) was significantly positive‐correlated with *Enterococcus* and *Weissella* (*p* < .001), and category E (amino acid transport and metabolism) had a significant positive association with *Staphylococcus* (*p* < .001) and a negative association with *Jeotgalicoccus* and *Sphingobacterium* (*p* < .001). Conversely, the correlation was very significantly negative between Category T and *Staphylococcus* and very significantly positive between Category T and *Bacillus* (*p* < .001). The fermentation of Douchi is actually the growth and metabolism process of microorganisms. Studies found that *Bacillus* had a significant positive correlation with gene sequences responsible for bacterial proliferation and defense, and a significant negative relation with sequences charging energy metabolism. The result complied with the physiological characteristics of *Bacillus*. Moreover, *Enterococcus* and *Weissella* can significantly boost transport and metabolism of carbohydrate, providing raw materials for subsequent energy creation. With these materials, *Providencia*, *Proteus*, *Vagococcus*, and *Sphingobacterium* and other dominant bacteria can produce energy for fermentation, and thus accelerate the process.

This paper also predicted the bacterial phenotype in Douchi samples (Figure S4). There were some yet insignificant differences between the two groups regarding to Oxygen Utilizing, Biofilm Forming, Contains, Mobile Elements, and Stress Tolerant, whereas big differences existed in Gram Positive, Gram Negative and Potentially Pathogenic. Further research on the two processes found that no significant difference in items such as Oxygen Utilizing was observed between the wet and dried Douchi. That was because both the water added in wet fermentation and water seal in dry fermentation after tank filling can isolate oxygen. However, it was worth noting that the pathogenic potential of microorganisms in dried Douchi samples was higher than that of wet Douchi. This difference may be derived from the community structure variance caused by microbial interactions during the fermentation process.

## 4 CONCLUSIONS

Microbial diversity in Douchi samples was interpreted using Illumina MiSeq high‐throughput sequencing. Results indicated that the dominant bacteria in samples were *Bacillus*, *Brevibacillus*, *Enterococcus*, *Proteus*, *Providencia*, *Staphylococcus*, *Ureibacillus,* and *Weissella*, and *Bacillus* can be used as biomarkers in dried samples. E‐nose analysis of flavor quality reported more intensive aromatic substances in dried samples, but significantly lower W6S and W3S value compared with the wet samples. Ten out of 18 dominant genera were strongly correlated with 7 flavor indicators. There was positive correlation between most bacteria genus and sensor indicators, among which *Brevibacillus* and *Aneurinibacillus* had significant positive correlations with three aromatic characteristics, while *Vagococcus* and *Corynebacterium* were negatively associated.

## CONFLICT OF INTEREST

The authors have declared no conflict of interest.

## Supporting information

Fig S1

Fig S2

Fig S3

Fig S4

Tab S1

## Data Availability

Sequences can be obtained by: https://www.mg‐rast.org/mgmain.html?mgpage=project&project=mgp96501
