# Supplementary figures and images for: Characterization of bacterial community and flavor differences of different types of Douchi
Source: Food Sci Nutr. 2021 May 18;9(7):3460–9. doi: 10.1002/fsn3.2280 (PMC8269581; doi:10.1002/fsn3.2280)

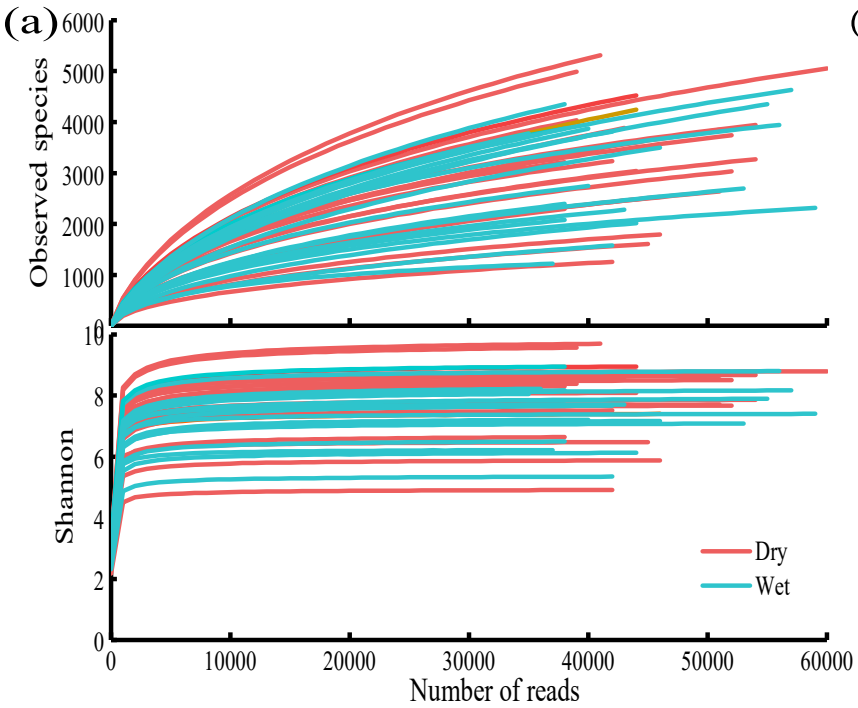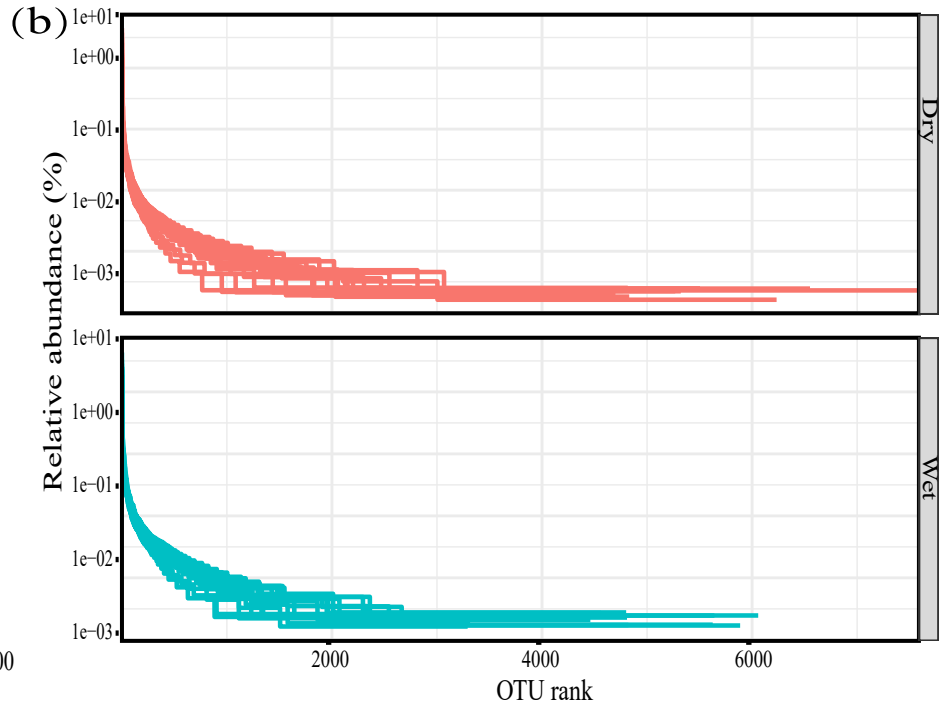

Supplement: Supplementary file 1 — Fig S1 [file FSN3-9-3460-s005.pdf]

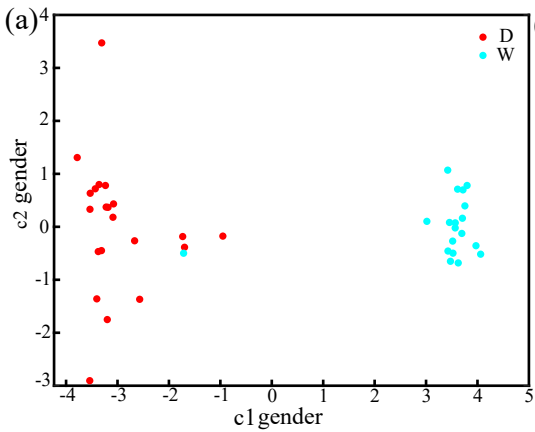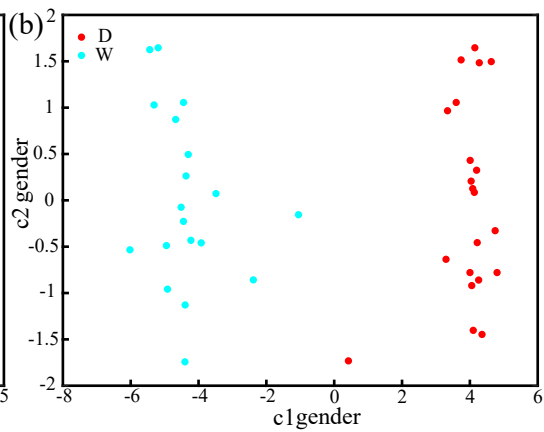

Supplement: Supplementary file 2 — Fig S2 [file FSN3-9-3460-s004.pdf]

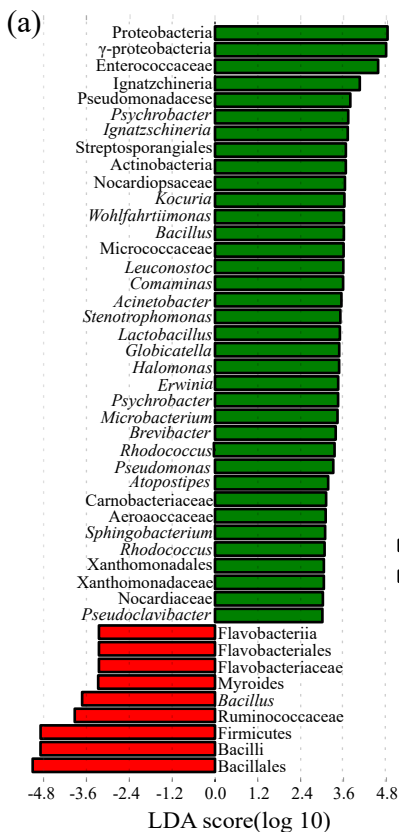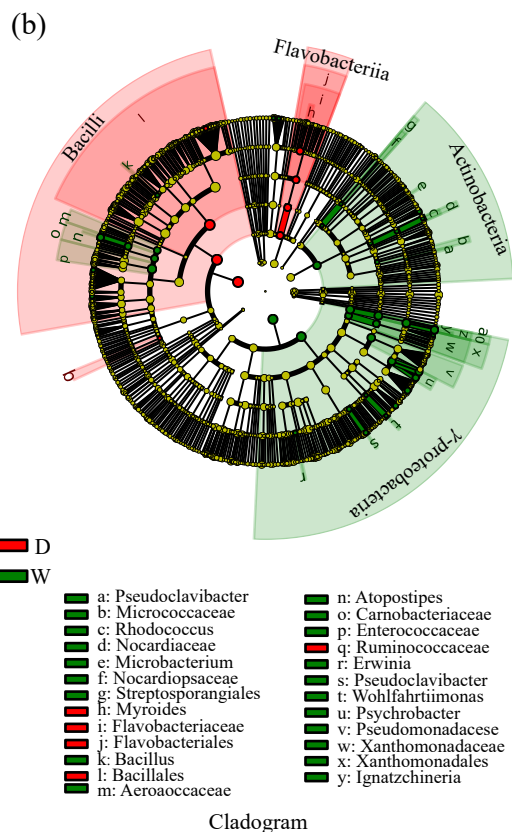

Supplement: Supplementary file 3 — Fig S3 [file FSN3-9-3460-s001.pdf]

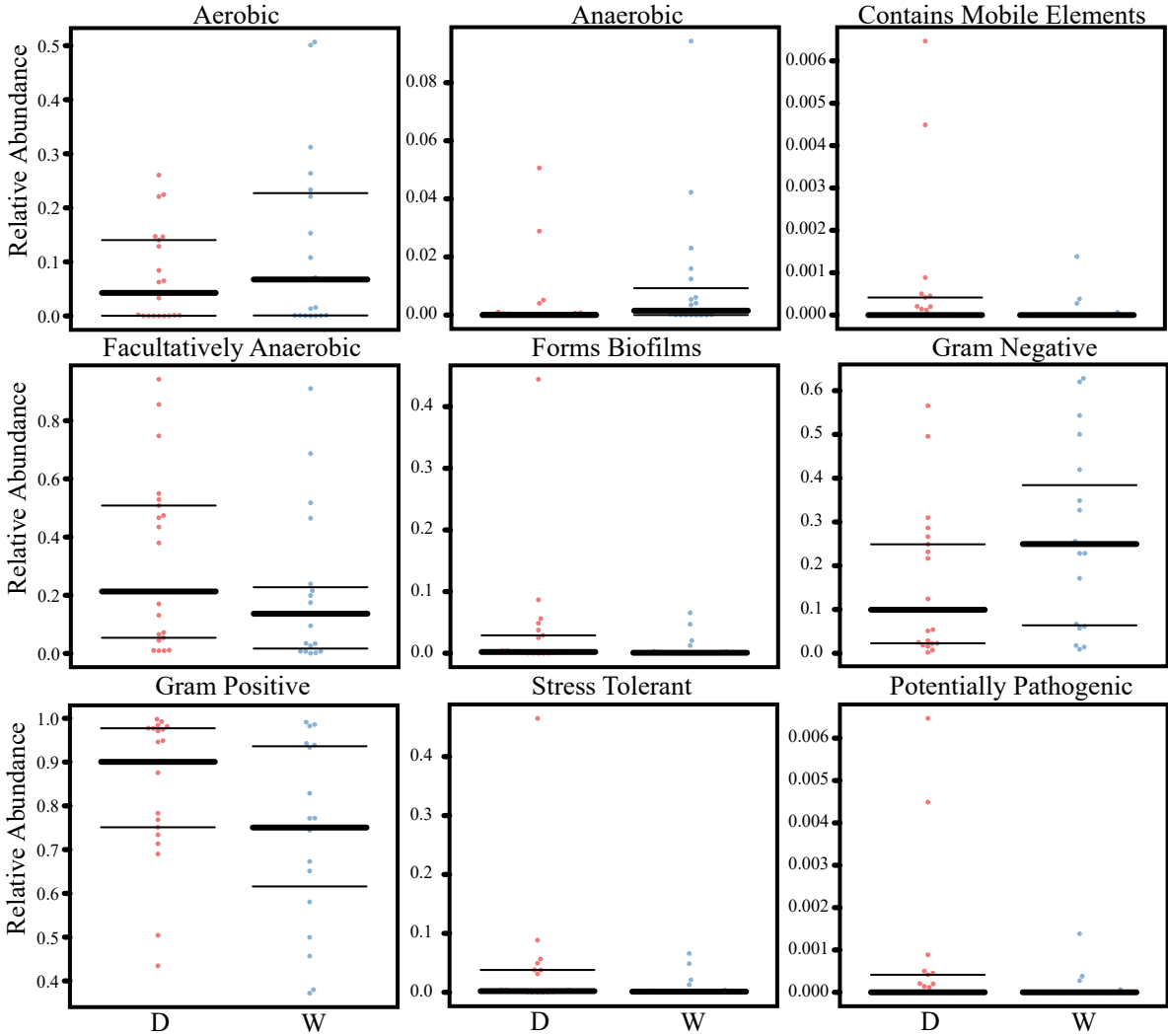

Supplement: Supplementary file 4 — Fig S4 [file FSN3-9-3460-s002.pdf]
