# Supplementary material for: Characterization of bacterial community and flavor differences of different types of Douchi
Source: Food Sci Nutr. 2021 May 18;9(7):3460–9. doi: 10.1002/fsn3.2280 (PMC8269581; doi:10.1002/fsn3.2280)
Supplement: Supplementary file 5 — Tab S1 [file FSN3-9-3460-s003.docx]

**Table S1.** General information of sequence and alpha diversity in Douchi samples

| **Samples** | **No. reads** | **No. OTU** | **Chao1*** | **Observed species*** | **Shannon*** | **Simpson*** |
| --- | --- | --- | --- | --- | --- | --- |
| D1 | 55711 | 4023 | 4817 | 8 | 0.95 | 3268.16 |
| D2 | 52961 | 3784 | 4634 | 8 | 0.96 | 3029.35 |
| D3 | 52905 | 3386 | 4131 | 8 | 0.97 | 2638.89 |
| D4 | 43571 | 1798 | 1848 | 5 | 0.78 | 1254.93 |
| D5 | 53357 | 4546 | 5490 | 9 | 0.98 | 3743.33 |
| D6 | 55536 | 4830 | 5674 | 9 | 0.98 | 3943.77 |
| D7 | 46279 | 2153 | 2468 | 6 | 0.96 | 1608.45 |
| D8 | 52653 | 4699 | 6498 | 9 | 0.99 | 3849.64 |
| D9 | 62430 | 6233 | 7486 | 9 | 0.97 | 5054.11 |
| D10 | 46989 | 3142 | 4034 | 7 | 0.97 | 2530.95 |
| D11 | 46992 | 2345 | 2659 | 6 | 0.85 | 1793.50 |
| D12 | 39910 | 4816 | 4225 | 8 | 0.98 | 3212.98 |
| D13 | 41091 | 6551 | 7561 | 10 | 0.99 | 4986.67 |
| D14 | 45301 | 5321 | 7037 | 9 | 0.98 | 4523.31 |
| D15 | 40755 | 5504 | 6426 | 8 | 0.97 | 4034.18 |
| D16 | 44019 | 7588 | 7781 | 10 | 0.99 | 5309.51 |
| D17 | 44627 | 4770 | 7020 | 9 | 0.98 | 3882.87 |
| D18 | 45260 | 5268 | 7470 | 9 | 0.99 | 4241.90 |
| D19 | 44183 | 4417 | 4502 | 8 | 0.92 | 3231.12 |
| D20 | 39311 | 2765 | 3848 | 7 | 0.90 | 2293.05 |
| D21 | 46448 | 4240 | 4518 | 8 | 0.98 | 3038.11 |
| W1 | 57749 | 4822 | 5540 | 9 | 0.98 | 3946.01 |
| W2 | 44972 | 2538 | 3368 | 6 | 0.90 | 2017.03 |
| W3 | 54156 | 3315 | 4273 | 7 | 0.93 | 2692.09 |
| W4 | 42640 | 2090 | 2529 | 5 | 0.80 | 1577.12 |
| W5 | 43745 | 2804 | 3560 | 8 | 0.98 | 2276.30 |
| W6 | 38765 | 3056 | 3870 | 8 | 0.98 | 2390.69 |
| W7 | 39728 | 2606 | 3277 | 6 | 0.88 | 2078.86 |
| W8 | 38312 | 1959 | 1459 | 6 | 0.93 | 1218.11 |
| W9 | 41619 | 3431 | 4128 | 7 | 0.93 | 2747.73 |
| W10 | 40676 | 5641 | 5588 | 9 | 0.99 | 3870.87 |
| W11 | 43661 | 4809 | 6246 | 8 | 0.94 | 3816.22 |
| W12 | 37012 | 4561 | 6273 | 8 | 0.96 | 3762.16 |
| W13 | 40646 | 6058 | 6782 | 8 | 0.97 | 4349.17 |
| W14 | 60919 | 3292 | 3179 | 7 | 0.97 | 2315.11 |
| W15 | 41184 | 4632 | 6913 | 8 | 0.93 | 3872.72 |
| W16 | 36166 | 4801 | 5963 | 8 | 0.94 | 3779.26 |
| W17 | 47891 | 4460 | 5763 | 7 | 0.93 | 3499.31 |
| W18 | 58742 | 5886 | 7225 | 8 | 0.96 | 4632.71 |
| W19 | 57101 | 5625 | 7124 | 8 | 0.97 | 4350.73 |

* The sequencing depth is 35010 reads.
